# Use of AI-based tools for healthcare purposes: a survey study from consumers’ perspectives

**DOI:** 10.1186/s12911-020-01191-1

**Published:** 2020-07-22

**Authors:** Pouyan Esmaeilzadeh

**Affiliations:** Department of Information Systems and Business Analytics, College of Business, Florida International University, Miami, FL 33199 USA

**Keywords:** Artificial intelligence (AI), AI medical devices, Clinical decision support, Perceived benefits, Perceived risks, Intention to use

## Abstract

**Background:**

Several studies highlight the effects of artificial intelligence (AI) systems on healthcare delivery. AI-based tools may improve prognosis, diagnostics, and care planning. It is believed that AI will be an integral part of healthcare services in the near future and will be incorporated into several aspects of clinical care. Thus, many technology companies and governmental projects have invested in producing AI-based clinical tools and medical applications. Patients can be one of the most important beneficiaries and users of AI-based applications whose perceptions may affect the widespread use of AI-based tools. Patients should be ensured that they will not be harmed by AI-based devices, and instead, they will be benefited by using AI technology for healthcare purposes. Although AI can enhance healthcare outcomes, possible dimensions of concerns and risks should be addressed before its integration with routine clinical care.

**Methods:**

We develop a model mainly based on value perceptions due to the specificity of the healthcare field. This study aims at examining the perceived benefits and risks of AI medical devices with clinical decision support (CDS) features from consumers’ perspectives. We use an online survey to collect data from 307 individuals in the United States.

**Results:**

The proposed model identifies the sources of motivation and pressure for patients in the development of AI-based devices. The results show that technological, ethical (trust factors), and regulatory concerns significantly contribute to the perceived risks of using AI applications in healthcare. Of the three categories, technological concerns (i.e., performance and communication feature) are found to be the most significant predictors of risk beliefs.

**Conclusions:**

This study sheds more light on factors affecting perceived risks and proposes some recommendations on how to practically reduce these concerns. The findings of this study provide implications for research and practice in the area of AI-based CDS. Regulatory agencies, in cooperation with healthcare institutions, should establish normative standard and evaluation guidelines for the implementation and use of AI in healthcare. Regular audits and ongoing monitoring and reporting systems can be used to continuously evaluate the safety, quality, transparency, and ethical factors of AI-based services.

## Background

Artificial Intelligence (AI) generally refers to a computerized system (hardware or software) that is able to perform physical tasks and cognitive functions, solve various problems, or make decisions without explicit human instructions [1]. A range of techniques and applications are under the broad umbrella of AI, such as genetic algorithms, neural networks, machine learning, and pattern recognition [2]. AI can replace human tasks and activities within a wide range of industrial, intellectual, and social applications with resulting impacts on productivity and performance. AI, as non-human intelligence programmed to complete specific tasks, can overcome some of the computationally intensive and intellectual limitations of humans [3]. For example, AI could be a computer application that is competent to solve a complicated business problem for managers. AI-enabled devices generate personalized recommendations to customers based on an analysis of a huge dataset. Thus, it is believed that AI could be smarter than the best humans and experts in any field [2]. The value of using AI tools is perceived based on the trade-off between possible benefit and risk as the benefit is higher than the risk, greater value of using the technology is perceived.

AI technology, including algorithmic machine learning and autonomous decision-making, creates new opportunities for continued innovation in different industries ranging from finance, healthcare, manufacturing, retail, supply chain, logistics, and utilities [4]. AI can be used in the form of clinical decision support (CDS) to support patient-specific diagnosis and treatment decisions and perform population-based risk prediction analytics [5]. Promoting AI-based services has become one of the focal points of many companies’ strategies [6]. The important changes made by AI have inspired recent studies to examine the impacts and consequences of the technology and to investigate the performance implications of AI. Though, this objective needs an in-depth understanding of the factors affecting the acceptance of AI-based services by potential users in different manufacturing and service fields. Previous studies highlight the importance of AI in healthcare, especially in medical informatics [7]. AI is able to provide improved patient care, diagnosis, and interpretation of medical data [8]. A study shows that AI technology used for breast cancer screening reduces human detection errors, but some of the interrelated ethical and societal trust factors, as well as reliance on AI, are yet to be developed [9]. The use of AI-driven recommendations in health care may be different from other sectors, mainly because of highly sensitive health information and high levels of consumers’ vulnerability to possible medical errors.

In April 2018, the FDA (Food and Drug Administration) authorized the first AI device to diagnose diabetic retinopathy without a physician’s help in the USA [10]. An increasing number of healthcare service companies are investing in the development of AI embedded in mobile health devices or health apps to improve patient safety, increase practice quality, enhance patient care management, and decrease healthcare costs. However, previous studies suggest that not all individuals are willing to accept the use of medical AI devices [10]. Successful implementation of AI-based systems requires a careful examination of users’ attitudes and perceptions about AI [5]. Thus, investing in AI technology without recognizing potential users’ beliefs and willingness to accept AI devices may lead to a waste of resources and/or even a loss of customers. This is especially true in the healthcare sector, where patient engagement is considered as one of the most critical determinants of healthcare quality. If individuals do not view interacting with a medical AI device as useful, they may demand interactions with physicians, and in turn, the AI-based devices may remain unused. Therefore, understanding the decision drivers and barriers that lead to acceptance or refusal of the use of AI-based devices in healthcare delivery is fundamental for healthcare providers and hospitals that plan to introduce and/or increase AI device presence during healthcare delivery.

Moreover, following previous studies, healthcare professionals still express fundamental concerns about the implementation of AI-based tools in care services [11]. There is a need for researchers to more efficiently understand the current challenges related to AI technologies and analyze the urgent needs of health systems to design AI-enabled tools that are able to address them. However, little is know about the antecedents of risk beliefs associated with the use of AI-based devices for treatments from the general public’s perspective. Theoretical and qualitative study results demonstrate some factors that contribute to risk beliefs and individuals’ withdrawal from using AI clinical devices [10]. But, empirical studies to examine the positive and negative sides of using AI in medicine from consumers’ perspectives are still scarce. Besides, the significance and generality of this value-based mindset, and its actual connection to the intention to use AI in health care, have not been investigated.

The value is estimated based on the trade-off of technology [12]. Most AI-related studies use various acceptance models (e.g., TAM, UTAUT) and do not include value perceptions (benefit and risk beliefs) associated with AI, which may lead to intention to use [3]. However, the value-based adoption model is viewed as a more appropriate approach to explain the behavior of service consumers by indicating that most consumers support new technologies based on their personal perceptions [13]. A comparative study also proposes that the value-based adoption model best explains consumer acceptance of AI-based intelligent products compared to other widely used technology acceptance theory (i.e., TAM, TPB, UTAUT) [14]. Thus, we expect that risk-benefit evaluations of AI technology in human-centered services (such as health care) significantly affect the use of AI clinical tools by individuals.

Currently, the issues related to AI-based tools in healthcare are still within the realm of research. However, it is widely believed that these systems will fundamentally change medical practice in the near future [15]. Historically, the medical sector does not integrate technology as fast as other industries. Moreover, without the involvement, cooperation, and endorsement of stakeholders (such as healthcare professionals and patients) and a robust legislative and regulatory framework, the integration of AI into current medical workflow could be very challenging. The main objective of this study is to examine how potential users (individuals) perceive benefits, risks, and use of AI-based devices for their healthcare purposes. The benefit perceptions and risk beliefs of prospective users may affect their future adoption of AI devices. Patients may not decide what tools healthcare professionals should use in their practice, but they can definitely highlight possible concerns, challenges, and barriers that may refrain them from supporting and using the tools implemented and promoted by clinicians.

Using value-based consideration to predict behavioral intention in this study acknowledges the distinctive nature of the healthcare sector compared to other less sensitive service work [11]. Extending the previously suggested AI acceptance model by including a value-based approach to the intention to use AI devices, we propose a model for health care to be used in academic and practical studies with an aim to statistically model acceptance of AI-based devices among potential users. In this study, we survey individuals’ acceptance of AI technology and identify the factors that determine the intention to use AI tools specifically in the context of health care. We categorize possible factors underlying risk beliefs associated with AI clinical tools as threefold: technological, ethical, and regulatory concerns.

There are different types of medial AI, and this study focuses on devices with AI-based CDS features. This research offers significant and timely insight into the applications of AI-based CDS in healthcare. The findings of this study will provide critical insights to researchers and managers on the determinants of individuals’ intention to use AI-based devices for healthcare delivery. Results imply that incompatibility with instrumental, technical, ethical, or regulatory values can be a reason for rejecting AI technology in healthcare. Multi-dimensional concerns associated with AI clinical tools may also be viewed as a cause of technostress, which occurs when an individual is unable to adapt to using technology [16]. In the future, it is the patient’s or customer’s right to choose AI-driven recommendations over human care or vice versa. Nevertheless, we propose that AI device developers and programmers can devise some practical strategies to anticipate possible concerns, and in turn, minimize risks (i.e., the subject of the concern) to encourage individuals to use devices with AI-based CDS for healthcare purposes.

## Methods

This study drew on the existing literature to measure the constructs included in the model, and minor changes were made to the instrument to fit the AI context. This study adapted items to measure constructs from existing scales developed by studies in the AI domain. The final measure items used in this study are listed in the Appendix. Table 1 shows the definition of constructs used in this study.

### Hypotheses development

**Fig. 1:**
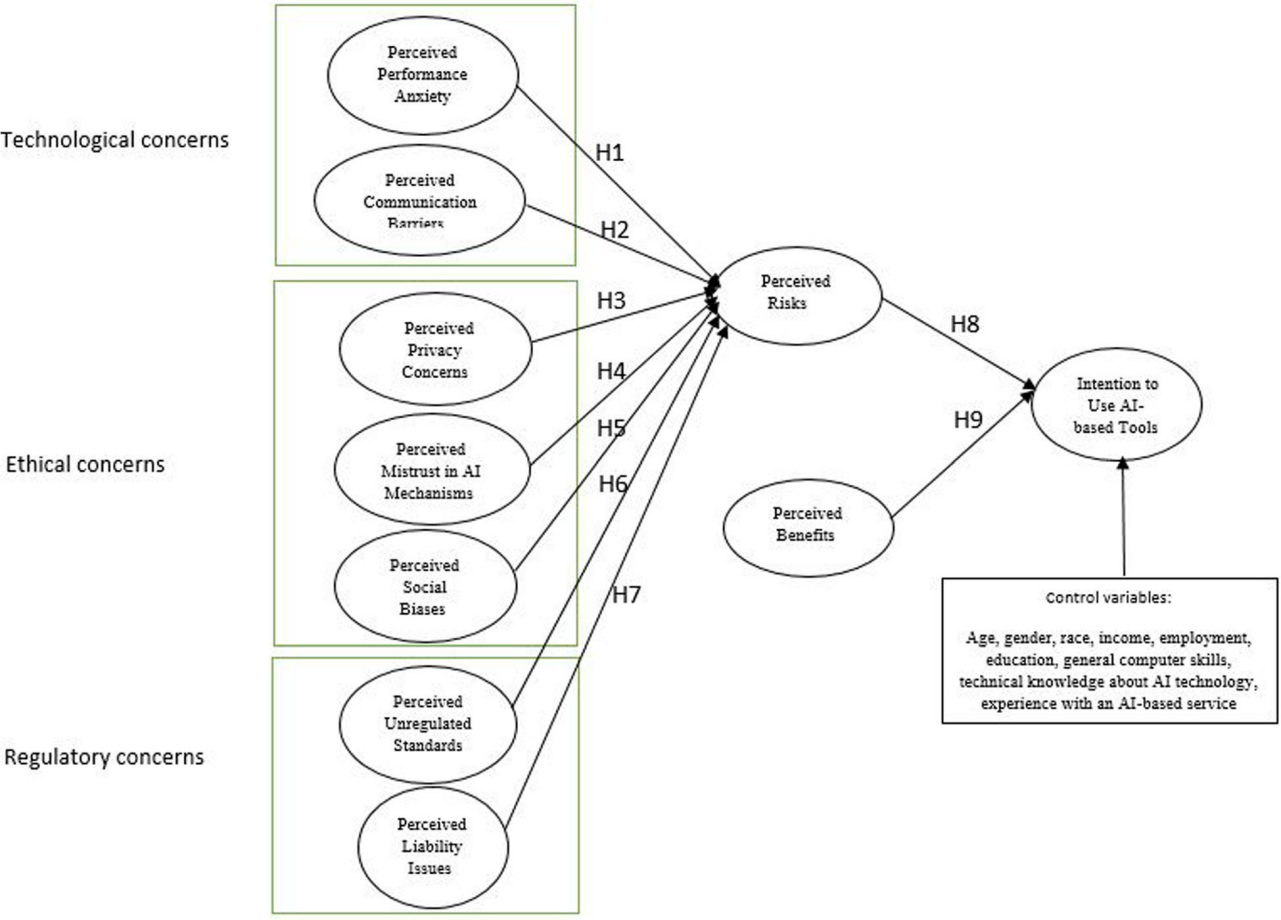
Research model

We bring perceived risks and its antecedents as well as perceived benefits together in a theoretical synthesis in which these concepts are seen to interact in ways that help shape the behavioral intention of AI users. The research model indicates that three concerns (technological, ethical, and regulatory) directly influence the general perceived risks associated with AI tools. Technological concerns include two dimensions: perceived performance anxiety and perceived communication barriers. Ethical concerns consist of three dimensions: perceived privacy concerns, perceived mistrust in AI mechanisms, and perceived social biases. Regulatory concerns comprise two dimensions: perceived unregulated standard and perceived liability issues. Moreover, both risk beliefs and benefit perceptions will influence individuals’ intention to use AI-based devices. In this study, we control for the effects of demographics and technology experience factors. The control variables are age, gender, race, income, employment, education, general computer skills, technical knowledge about AI technology, and experience with an AI-based service, which are found and tested by prior research as factors affecting the adoption of AI devices e.g., [3]. Figure 1 shows the proposed research model.

**Table 1:** Operationalization of variables

Construct	Construct definition	Source
Perceived performance anxiety	The degree to which an individual believes that AI-based tools and their features exhibit pervasive technological uncertainties.	Sarin, Sego [17]
Perceived communication barriers	The degree to which an individual feels that AI devices may reduce human aspects of relations in the treatment process.	Lu, Cai [18]
Perceived social biases	The degree to which a person believes that data used in the AI devices may lead to societal discrimination to a certain patient group (e.g., minority groups).	Reddy, Allan [19]
Perceived privacy concerns	The extent to which individuals concern about how AI-based devices collect, access, use, and protect their personal information	Stewart and Segars [20]
Perceived mistrust in AI mechanisms	The degree to which an individual believes that AI models and AI-driven diagnostics and recommendations in health care are still not trustworthy.	Luxton [21]
Perceived unregulated standard	The extent to which an individual believes that regulatory standards and guidelines to assess AI algorithmic safety are yet to be formalized.	Cath [22]
Perceived liability issues	The extent to which an individual is concerned about the liability and responsibility of using AI clinical tools.	Laï, Brian [10]
Perceived risks	The extent to which an individual believes that, in general, it would be risky for patients to use AI-based tools in health care.	Bansal, Zahedi [23]
Perceived benefits	The extent to which an individual believes that AI-based tools can improve diagnostics and care planning for patients.	Lo, Lei [24]
Intention to use AI-based tools	The extent to which an individual is willing to use AI-based services for diagnostics and treatments.	Turja, Aaltonen [11]

### Technological concerns

Technological concerns include the nature of diagnostic tasks, lack of transparency of AI process, safety of AI-driven recommendations, complexities in interpreting results, and issues with AI-user interaction architecture [25]. In this study, we consider two dimensions for technical concerns: perceived performance anxiety and perceived communication barriers.

### Perceived performance anxiety

Perceived performance anxiety refers to users’ perception of the likelihood that an IT system malfunctions, does not work as intended, and become unable to deliver the desired services [26]. AI-related studies consider the safety and quality of autonomous operations an essential factor affecting the use of AI devices [27]. According to Mitchell [28], AI systems are still vulnerable in many areas, such as hacker attacks. Hackers can change text files or images which may not have a human cognitive effect but could cause potentially catastrophic errors. Since the AI program may not understand the input and outputs, they are susceptible to unexpected errors and untraceable attacks. Consequently, these medical errors could endanger patient safety and result in death or injuries. Thus, users may become concerned that the mechanisms used by AI-based devices could lead to incorrect diagnoses or wrong treatments. A study indicates that incomplete and nonrepresentative datasets in AI models can produce inaccurate predictions and medical errors [29]. Thus, we propose that individuals may consider that possible functional errors resulting from the use of AI devices could lead to more risks.

H1: Perceived performance anxiety positively influences perceived risks.

### Perceived communication barriers

The use of AI devices in service delivery (such as healthcare) may cause noteworthy communication barriers between customers and AI devices [18]. Reliance on AI clinical tools may reduce the interactions and conversation between physicians and patients [30]. Consumers may refuse to use AI devices because of their need for human social interaction during service encounters [3]. AI technology fundamentally changes the traditional physician-patient communications [31]. Thus, individuals may worry as they may lose face-to-face cues and personal interactions with physicians. AI causes challenges to patient-clinician interactions, as clinicians need to learn how to interact with the AI system for healthcare delivery, and patients are required to reduce the fear of technology [32]. As AI continues to proliferate, users still encounter some challenges about the effective use of AI, such as how the partnership between AI systems and humans could be synergic? [2]. A study proposes that more sophisticated technologies should be integrated into current medical AI systems to improve human-computer interactions and streamline the flow of information between two parties [25]. Therefore, AI tools may reduce conversation between physicians and patients, and it may emerge more risk beliefs. We develop the second hypothesis as follows:

H2: Perceived communication barriers positively influence perceived risks.

### Ethical concerns

Ethical concerns include trust issues about AI and human behavior, compatibility of machine versus human value judgment, moral dilemmas, and AI discrimination [25]. In this study, we consider three dimensions for ethical concerns: perceived privacy concerns, perceived mistrust in AI mechanisms, and perceived social biases.

### Perceived privacy concerns

Health-related data is one of the most sensitive information about a person [30]. In healthcare services, respecting a person’s privacy is an essential ethical principle because patient privacy is associated with wellbeing and personal identity [22]. Thus, patients’ confidentiality should be respected by healthcare providers by protecting their health records, preventing secondary use of data, and developing a robust system to obtain informed consent from them for healthcare purposes [33]. If the privacy needs of patients are not met, patients will be affected by psychological and reputational harm [34]. Data breaches would increase risk believes associated with AI models designed to share personal health information. There is a concern that anonymized data can be reidentified through AI processes, and this anxiety may exacerbate privacy invasion and data breach risks [19].

AI technology in public health requires large datasets. Thus, collection, storage, and sharing of medical data raise ethical questions related to safety, governance, and privacy [35]. Privacy is one of the most critical concerns while using AI systems because users’ personal data (such as habits, preferences, and health records) is likely to be stored and shared across the AI network [25]. Method of data collection for AI may increase risks as AI systems need huge datasets, and patients are concerned that their personal information will be collected without their knowledge [30]. Accordingly, the next hypothesis is proposed as follows:

H3: Perceived privacy concerns positively influence perceived risks.

### Perceived mistrust in AI mechanisms

Trust between public and healthcare systems is essential for effective healthcare delivery [36]. Gaining the trust of the general public in the use of AI in healthcare is considered as an important challenge to the successful implementation of AI in medical practices [37]. Perceived mistrust in AI mechanisms refers to users’ perception that AI’s predictive and diagnostic models are not trustworthy [19]. A study reports that, in general, individuals are likely to exhibit a lack of trust in the features of AI systems [38]. For instance, people may not trust AI ‘s predictive power and diagnostic ability for treatment purposes. Lee, Kim [39] indicate that the autonomy of AI systems affects the users’ perception of trustworthiness. Trust in AI-based tools (such as AI medical devices) is found to be a significant factor affecting adoption decisions [40]. When patients cannot understand the inside workings of AI devices, they may exhibit lower trust in their functions and how they generate treatment solutions and recommendations. The nature of AI models (such as deep learning) may increase a lack of transparency related to AI systems and threaten patient trust, which in turn, result in greater risk belives [37]. Therefore, we hypothesize:

H4: Perceived mistrust in AI mechanisms positively influences perceived risks.

### Perceived social biases

Studies in other contexts show that AI models overestimate the risk of crime among members of a certain racial group [41]. In the healthcare context, biased AI models may overestimate or underestimate health risks in specific patient populations. For instance, AI systems may engage in stereotyping and exhibit gender or racial bias. Bias in AI models my also occur when datasets are not representative of the target population, or incomplete and inaccurate data are used by AI systems for decision-making [30]. Societal discrimination (such as poor access to healthcare) and small samples (such as minority groups) can lead to unrepresentative data and AI bias [19]. A study reports that the current architecture of AI systems needs a more sophisticated structure to understand human moral values [42]. If the AI algorithm is not transparent, it may exhibit some levels of discrimination, even though humans are not involved in the decision-making process [25]. The main purpose of AI is to create an algorithm that functions autonomously to find the best possible solutions to questions [43]. However, researchers argue that predictive programs can be inevitably biased due to an overrepresentation of the social minorities in the pattern recognition process [44]. Some studies support this argument by showing that AI algorithms may be coded biasedly, which can produce racist decisions [45]. Therefore, if people are concerned that AI devices could lead to morally flawed practices in healthcare by overestimating or underestimating health risks in a certain patient population, they become more likely to perceive more risks associated with AI. This discussion results in the following hypothesis:

H5: Perceived social biases positively influence perceived risks.

### Regulatory concerns

Regulatory concerns include governance of autonomous AI systems, responsibility and accountability, lack of rules of accountability in the use of AI, and lack of official industry standards of AI use and performance evaluation [25]. In this study, we consider two dimensions for regulatory concerns: perceived unregulated standards and perceived liability issues.

### Perceived unregulated standards

Regulatory concerns are found as critical challenges to the use of AI in healthcare as policies and guidelines for AI tools are not transparent yet [22]. Existing literature indicates that regulatory agencies require to agree on a set of standards that medical AI rollout must be rated against. For instance, how decent is the auditability of decisions made by autonomous AI-based devices? [25]. Due to the intelligence nature of AI systems, regulatory agencies should establish new requirements, official policy, and safety guidelines regarding AI rollout in healthcare [10]. For example, there is a legal need to evaluate the decision made by AI systems in case of litigation. AI tools operate based on the auto-learn models, which improve their performance over time [46]. This inner mechanism differentiates AI-based devices from other tools in healthcare and gives rise to new regulatory concerns that may not be a case in other domains. Generally, algorithms that change continuously with features that are not limited to the original accepted clinical trials may need a new range of policies and guidelines [30]. Regulatory authorities are yet to formalize standards to evaluate and maintain the safety and impact of AI in many countries [19]. Thus, people may become concerned if an appropriate regulatory and accreditation system regarding AI-based devices is not in place yet. The lack of clear guidelines to monitor the performance of AI tools in the medical context can lead to higher risk beliefs associate with AI. We propose the following hypothesis:

H6: Perceived unregulated standards positively influence perceived risks.

### Perceived liability issues

Accountability and liability are another concern related to AI. Previous studies in public health demonstrate legal concerns about who will account for AI-based decisions when errors occur using AI systems [47]. Wirtz, Weyerer [48] emphasize the challenges connected to the responsibility and accountability of AI systems. It is still not clear how the regulatory concerns around responsibility and accountability of using solutions made by AI systems can be dealt with formally [25]. As AI-based devices in healthcare make autonomous decisions, the accountability question becomes very hard to answer. For instance, it will create a risky situation for both clinicians and patients when it is still not clear who becomes responsible if AI-based tools offer wrong recommendations in healthcare [29]. Liability complexity becomes higher since it is not transparent to what extent AI systems are able to guide and control clinical practices [49]. Responsibility concerns are not only limited to the incidents that AI may generate errors. Another aspect of liability risk refers to the situation where appropriate treatment options recommended by AI are mistakenly dismissed [21]. Thus, the higher the perceived liability issues, the greater the risk beliefs associated with AI will be. We pose the next hypothesis as follows:

H7: Perceived liability issues positively influence perceived risks.

### Perceived risks

The risk perceptions related to an IS system can reduce the possible utility attached to the technology [50]. As AI-based devices are not in line with traditional medical practices, the ambiguity about the safety and efficacy of AI models in healthcare are still strong reasons that facilitate users’ risks [51]. Since the nature of diagnostic tasks causes a lack of transparency, current AI systems used in healthcare are considered as a black box to users, which acts as a barrier to the adoption of AI technology [52]. If the degree of uncertainty associated with the use of AI-based tools is high, individuals are less likely to use them in the future. The general risk of using AI-based devices for medical purposes exacerbates individual intention to adopt AI. The higher the potential loss associated with the use of AI devices, the lower the people’s willingness to use them. Thus, we propose:

H8: Perceived risks negatively influence individuals’intention to use AI-based tools.

### Perceived benefits

AI can be used in healthcare for risk prediction and recommendation generation. Big data and AI significantly improve patient health based diagnosis and predictive capability [53]. Recent studies show new opportunities for AI applications within medical diagnosis and pathology where medical tasks can be done automatedly with higher speed and accuracy [52]. AI can improve different aspects of healthcare delivery, such as diagnostics, prognosis, and patient management [19]. For instance, AI is shown to diagnose skin cancer more efficiently than dermatologists [54]. A study demonstrates that hedonic aspects, such as enjoyment and curiosity about AI technology, are stronger in predicting behavioral intention to use AI products than utilitarian aspects (e.g., usefulness) [14]. This point does not hold in the healthcare context since AI systems are mainly used in healthcare for utilitarian aspects such as patient-specific diagnosis, treatment decision making, and population risk prediction analysis [55]. Thus, in the benefit perceptions, we only focus on utilitarian aspects, not other motivational factors. Sun and Medaglia [38] identify the lack of sufficient knowledge of the AI technologies’ values and advantages as potential barriers to the adoption of AI systems. Individuals will endorse and use AI clinical tools if they believe that AI will bring essential benefits to their healthcare delivery. The higher the perceived benefits from AI-based devices, the higher the individuals’ intention to use them in the future. Thus, we develop the last hypothesis as follows:

H9: Perceived benefits positively influence individuals’ intention to use AI-based tools.

### Pilot test

Once the initial questionnaire was developed, we consulted five professionals in the AI domain to improve the content validity of our study and finalize the AI definitions, the mechanisms of AI-based CDS, and the questions used in this study. Consistent with the experts’ suggestions, we modified the terms used to define AI and improved the scenario and questions to ensure that they were transparent enough and easy to understand for the public. Then, we conducted a face validity with 15 students (6 Ph.D. and 9 Master’s degree in IS) to ensure that the readability and wording of the questions were acceptable and consistent with the objectives of our study. Thus, we reworded some ambiguous terms and removed technical language and jargon to describe the scenario most understandably and straightforwardly. Finally, prior to the main data collection, we conducted a pilot test with 117 undergraduate students at a large Southeastern university in the United States to ensure that the instrument had acceptable reliability and validity. We computed the Cronbach’s alpha for each construct (i.e., perceived benefits α = 0.94, perceived riks α = 0.90, performance risks α = 0.91, perceived social biases α = 0.88, perceived privacy concerns α = 0.94, perceived mistrust in AI mechanisms in AI mechanisms α = 0.92, perceived communication barriers α = 0.93, perceived unregulated standard α = 0.94, perceived liability issues α = 0.94, intention to use AI-based devices α = 0.94). All Cronbach’s alpha values were above the cutoff point of 0.7, indicating that the instrument was internally consistent [56].

### Data collection

Data were collected in April 2020 from Amazon’s Mechanical Turk (MTurk) to obtain a representative group of subjects in the United States. MTurk is a survey tool that has been used in several studies as an acceptable means to collect individual-level data from the general population of interest [57]. Studies highlight that recruiting survey respondents from MTurk can improve the reliability of data compared to traditional subject pools [58]. Researchers as requesters can use this crowdsourcing website to reach out to potential subjects (i.e., MTurk workers) in numerous countries to conduct a survey. MTurk workers with an Amazon account can perform a task (such as participating in a study) in exchange for a monetary payment.

Since AI may not be considered as a routine technology for many individuals, a detailed description of AI was provided at the beginning of the online survey to ensure that respondents completely comprehended the context and purpose of the study. Moreover, since the focus of this study is on AI-based CDS, we defined a scenario about AI-supported devices with CDS features used for health care purposes. In the scenario, we described a situation in which individuals have the option of using an AI-based device when they are suffering from a disease. The steps of using AI technology are clearly explained to respondents. For instance, in case of feeling sick, they can directly enter their signs, symptoms, and critical health complaints into the AI-based device. Their health information will be recorded in a big database. Then, the AI system analyzes their health data and compares them to the learned patterns (for example, the list of diseases and medicines) and draws some clinical conclusions. Finally, based on the pattern found, AI creates a report including some diagnostic options, some treatment choices, prescription advice (e.g., dose, frequency, and name of medications they need to take), care planning (e.g., resting at home, taking suggested medicines for a specific period or visiting a professional immediately). In summary, we highlighted that the devices with AI-based CDS are able to analyze clinical data and make medical decisions for patients without direct physician interactions. It should be mentioned that the definitions and given scenario were illustrated in a way in which they are understandable for the general public.

After reading the scenario, respondents were asked to reflect their perceptions about possible risks, potential benefits, and intention to use devices with AI-based features in the future. Since the data collection was performed anonymously, respondents only entered their data related to the main variables of interest and some standard demographic variables (such as age, race, gender, and age), but their names or any identification numbers were not requested in the survey. The incentive for participation was a monetary reward ($2).

### Analyses

Totally, in a month, 500 individuals completed the survey (surveys with incomplete answers were discarded). As mentioned in previous studies, one general concern in data collection is a potential lack of attention and random responses [59]. Consistent with other studies, we used “captcha” questions to prevent and identify careless, hurried, or haphazard answers [60]. For instance, in the captcha questions, the respondents were presented with a challenge (such as reverse coding questions) to capture whether they completed the survey carelessly or in a hurry. Based on answers to these verification questions, seventy-three responses were dropped. This ratio is similar to those reported in previous studies that used MTurk for data collection [58]. Thus, concerns that online respondents might reply randomly or haphazardly to complete the survey quickly were alleviated. After excluding responses that failed the response quality questions, the final set of useable and valid responses contained 427 samples. We also used Mplus to assess the power of analysis and determine the sample size [61]. Given the number of observed and latent variables in the model, the anticipated effect size (0.3), the desired probability (0.8), and statistical power levels (α = 0.05 and power β = 0.95), the minimum sample size for the model structure is 400. Therefore, this study is adequately powered, as 427 samples could be sufficient to reduce possible sampling errors and minimize Type II errors. This is consistent with both the ratio of indicators to the latent variables approach and the function of minimum effect, power and significance suggested by Westland [62]. In this study, data are analyzed with IBM SPSS AMOS (version 26) in order to test the hypotheses within a Structural Equation Modeling (SEM) framework.

Before data were statistically analyzed, normality was evaluated as this is important for distributions of data to exhibit this trait, to facilitate unbiased and consistent models [63]. According to Hair, Black [64], skewness expresses the symmetry while kurtosis explains the peakedness of distributions. Thus, all the constructs used in the model were scrutinized against the normality assumptions. An examination of the skewness and kurtosis of the constructs showed a skewness range from − 0.068 to 0.003, and a kurtosis range from − 1.192 to − 0.216. Based on these findings, all the values fall within the prescribed limit and maximum acceptable levels of 2 for skewness and 7 for kurtosis tests [65].

To validate the survey instrument, we performed a Confirmatory Factor Analysis (CFA) on all the constructs to assess the measurement model. IBM SPSS AMOS (version 26) was used to test convergent validity and discriminant validity. According to Gefen, Straub [66], convergent validity can be tested by examining the standardized factor loading, composite reliability, and the Average Variance Extracted (AVE). Table 2 shows the results of convergent validity test. All values of composite reliabilities were more than the threshold value of 0.7, which highlighted that the reliability of constructs was adequate [67]. According to Hair, Black [64], a factor loading of 0.7 or greater is acceptable. In this study, all reported standardized factor loadings were greater than 0.7. The AVE of each construct was calculated using standardized factor loadings. All reported values of the AVE were also greater than 0.5, which met the minimum requirement [68]. These measures indicated that the convergent validity of the measurement model was acceptable.

**Table 2:** Results of convergent validity

Construct	Items	Standardized Factor loading (> 0.7)	Composite reliability (> 0.7)	AVE (> 0.5)
Perceived Performance Anxiety	PPA1	0.86	0.924	0.709
	PPA2	0.86		
	PPA3	0.85		
	PPA4	0.80		
	PPA5	0.84		
Perceived Social Biases	PSB1	0.80	0.919	0.694
	PSB2	0.84		
	PSB3	0.88		
	PSB4	0.78		
	PSB5	0.86		
Perceived Privacy Concerns	PPC1	0.80	0.952	0.767
	PPC2	0.89		
	PPC3	0.90		
	PPC4	0.87		
	PPC5	0.92		
	PPC6	0.87		
Perceived Mistrust in AI Mechanisms	PMT1	0.87	0.938	0.751
	PMT2	0.85		
	PMT3	0.89		
	PMT4	0.89		
	PMT5	0.83		
Perceived Communication Barriers	PCB1	0.87	0.934	0.738
	PCB2	0.87		
	PCB3	0.88		
	PCB4	0.90		
	PCB5	0.77		
Perceived Unregulated Standards	PUS1	0.86	0.944	0.771
	PUS2	0.89		
	PUS3	0.88		
	PUS4	0.90		
	PUS5	0.86		
Perceived Liability Issues	PL1	0.89	0.945	0.742
	PL2	0.86		
	PL3	0.90		
	PL4	0.86		
	PL5	0.86		
	PL6	0.76		
Perceived Benefits	PB1	0.84	0.943	0.705
	PB2	0.85		
	PB3	0.89		
	PB4	0.84		
	PB5	0.85		
	PB6	0.74		
	PB7	0.86		
Perceived Risks	PR1	0.8	0.910	0.670
	PR2	0.85		
	PR3	0.84		
	PR4	0.82		
	PR5	0.78		
Intention to Use AI-based Tools	INT1	0.83	0.940	0.758
	INT2	0.87		
	INT3	0.90		
	INT4	0.89		
	INT5	0.86		

We also tested the discriminant validity of the constructs (Table 3). All the diagonal values (the square roots of the AVEs) were greater than 0.7 and exceeded the correlations between any pair of constructs [69]. Therefore, the result indicates that the model fulfills the requirements of discriminant validity, and it is assumed that the model also has adequate discriminant validity.

**Table 3:** Results of discriminant validity

Construct	Mean	SD.	PPA	PSB	PPC	PT	PCB	PUS	PL	PB	PR	INT
PPA	3.48	0.99	**0.842**									
PSB	3.19	1.03	0.499	**0.833**								
PPC	3.41	1.10	0.437	0.439	**0.875**							
PMT	3.14	0.98	−0.328	−0.177	−0.127	**0.866**						
PCB	3.62	1.08	0.377	0.403	0.320	−0.168	**0.859**					
PUS	3.67	1.04	0.437	0.438	0.446	−0.160	0.459	**0.878**				
PL	3.69	1.06	0.455	0.396	0.399	−0.235	0.555	0.526	**0.861**			
PB	3.76	1.03	−0.186	− 0.137	− 0.048	0.495	− 0.045	0.049	− 0.045	**0.839**		
PR	3.49	0.96	0.451	0.464	0.515	−0.478	0.596	0.522	0.553	−0.272	**0.818**	
INT	3.33	1.08	−0.338	−0.192	− 0.158	0.453	− 0.214	−0.156	− 0.220	0.610	− 0.338	**0.870**

Table legend: *PPA* Perceived Performance Anxiety, *PSB* Perceived Social Biases, *PPC* Perceived Privacy Concerns, *PMT* Perceived Mistrust in AI Mechanisms, *PCB* Perceived Communication Barriers, *PUS* Perceived Unregulated Standards, *PL* Perceived Liability Issues, *PB* Perceived Benefits, *PR* Perceived Risks, *INT* Intention to Use AI-based Tools

Although the correlations among constructs were not highly noticeable, we checked for multicollinearity by computing the Variance Inflation Factor (VIF) and tolerance values for the predictor variables. The resultant VIF values were between 1.79 and 2.64, which were below the cutoff value of 5, and the tolerance values were in the range of 0.55 and 0.37, which were higher than the threshold of 0.1 [56]. Thus, multicollinearity is not an issue in this research. Finally, as using the self-report survey method can raise the common method variance issue, we examined the potential for common method bias [70]. We conducted Harman’s one-factor test to check if the common method bias would be a problem [71]. All factors together could explain 75.40% of the total variance, while none of the factors accounted for most of the covariance among measures (< 20%). Accordingly, test results implied that common method bias was a non-significant threat in our sample.

### Control variables

Factors that do not represent the core variables (i.e., those included in the causal model) of this study, but which may affect the inter-relationships between the core variables, have been controlled. As mentioned previously, we controlled age, gender, race, income, employment, education, general computer skills, technical knowledge about AI technology, and experience with an AI-based service. Although the causal model seems to represent individuals’ intention to use AI-based devices, we found that the effects of control variables were not negligible. Based on the findings, gender (ß = − 0.12, *p* < 0.05), annual household income (ß = 0.11, *p* < 0.05) education level (ß = 0.14, *p* < 0.01), employment (ß = 0.12, *p* < 0.05), technical knowledge about AI technology (ß = 0.13, *p* < 0.01) and familiarity with an AI-based service (ß = 0.22, *p* < 0.001) influence intention to use. These results imply that employed male users with higher education levels, higher annual household income, advanced technical knowledge about AI, and greater experience with AI may exhibit a higher intention to use AI technology for healthcare purposes. However, no effects of age, race, and general computer skills were found on the intention to use.

## Results

### Descriptive statistics

Table 4 depicts the respondents’ characteristics. The demographic characteristics show that the majority of respondents were white (69.7%) with a full-time job (64.5%). The gender was equally distributed with males (51.1%) and females (48.9%). Respondents were fairly young as 67.1% of them were younger than 40 years old. Around 73% of respondents had some college, 2-year old degree, or bachelor’s degree. About 65% had an annual household income between $25,000 and less than $100,000. 41% of participants reported that they used an AI-based service for other reasons not related to healthcare (such as financial decision making), and around 55% declared that they were moderately or very familiar with general AI-based devices. Only 23% used an AI-enabled health service (such as AI embedded in smart medical devices), and 60% of them were either not familiar or slightly familiar with the AI applications in healthcare. Regarding general computer literacy, 80% indicated that their computer skills were good or excellent, and 74% rated their technical knowledge about AI average or good. Finally, 70% of respondents reported that their general health literacy was good or excellent. We can interpret that, although most of the respondents did not experience an AI-based device for healthcare purposes, they were familiar with general AI tools (for other purposes) either through direct experience, reading articles, following the news, or social media activities.

**Table 4:** Sample characteristics

Variable	Categories	Percentage (%)
Gender	Male	51.1
	Female	48.9
Age	Under 20	0.7
	20–29	29.6
	30–39	36.8
	40–49	17.6
	50–59	8.5
	60 or older	6.8
Annual household income	<$25,000	15.6
	$25,000–$49,999	24.8
	$50,000–$74,999	23.1
	$75,000–$99,999	17.3
	$100,000- -$150,000	14.3
	More than $150,000	4.9
Education	Less than high school	1
	High school graduate	9.1
	Some college	15
	2-year degree	11.1
	Bachelor’s degree	47.2
	Master’s degree	13.4
	Doctorate	3.3
Employment status	Employed- full time	64.5
	Employed-part time	15.3
	Unemployed	11.1
	Retired	3.9
	Student	5.2
Race/ethnicity	White	69.7
	African American	8.8
	Asian	15
	Hispanic	4.6
	Mixed	1.6
	Other	0.3
Have you ever used any AI-enabled services or devices for any reason except for healthcare? (Such as AI embedded in smart devices for any purposes such as financial decision-making)	Yes	41
	No	59
Generally, how familiar are you with an AI-based device (used for any purposes except for healthcare)?	Not familiar at all	10.7
	Slightly experienced	26.1
	Moderately experienced	37.1
	Very experienced	18.2
	Extremely experienced	7.8
Have you ever used any AI-enabled health services? (Such as AI embedded in smart medical devices)	Yes	23.5
	No	76.5
How familiar are you with these AI-based devices used for clinical purposes?	Not familiar at all	30.6
	Slightly experienced	30.0
	Moderately experienced	22.8
	Very experienced	8.8
	Extremely experienced	7.8
Have you ever experienced a data breach incident (i.e.., data loss, including personal, health, or financial information)?	Yes	32.7
	No	67.3
Overall, do you think your health information is .....?	Sensitive	74.9
	Non-sensitive	16.0
	No idea	9.1
How do you generally rate your computer skills?	Terrible	0.7
	poor	0,7
	average	18.6
	Good	45.0
	Excellent	35.2
How do you rate your technical knowledge about AI?	Terrible	2.0
	poor	14.3
	average	49.5
	Good	24.8
	Excellent	9.4
How did you gather information about general AI tools?	Articles in magazines/newspapers	43.6
	Social media	35.1
	Friends and family	16.7
	Technical books	4.6
How do you rate your health literacy?	Terrible	0.3
	poor	2.9
	average	26.7
	Good	47.2
	Excellent	22.8

### Structural model

**Fig. 2:**
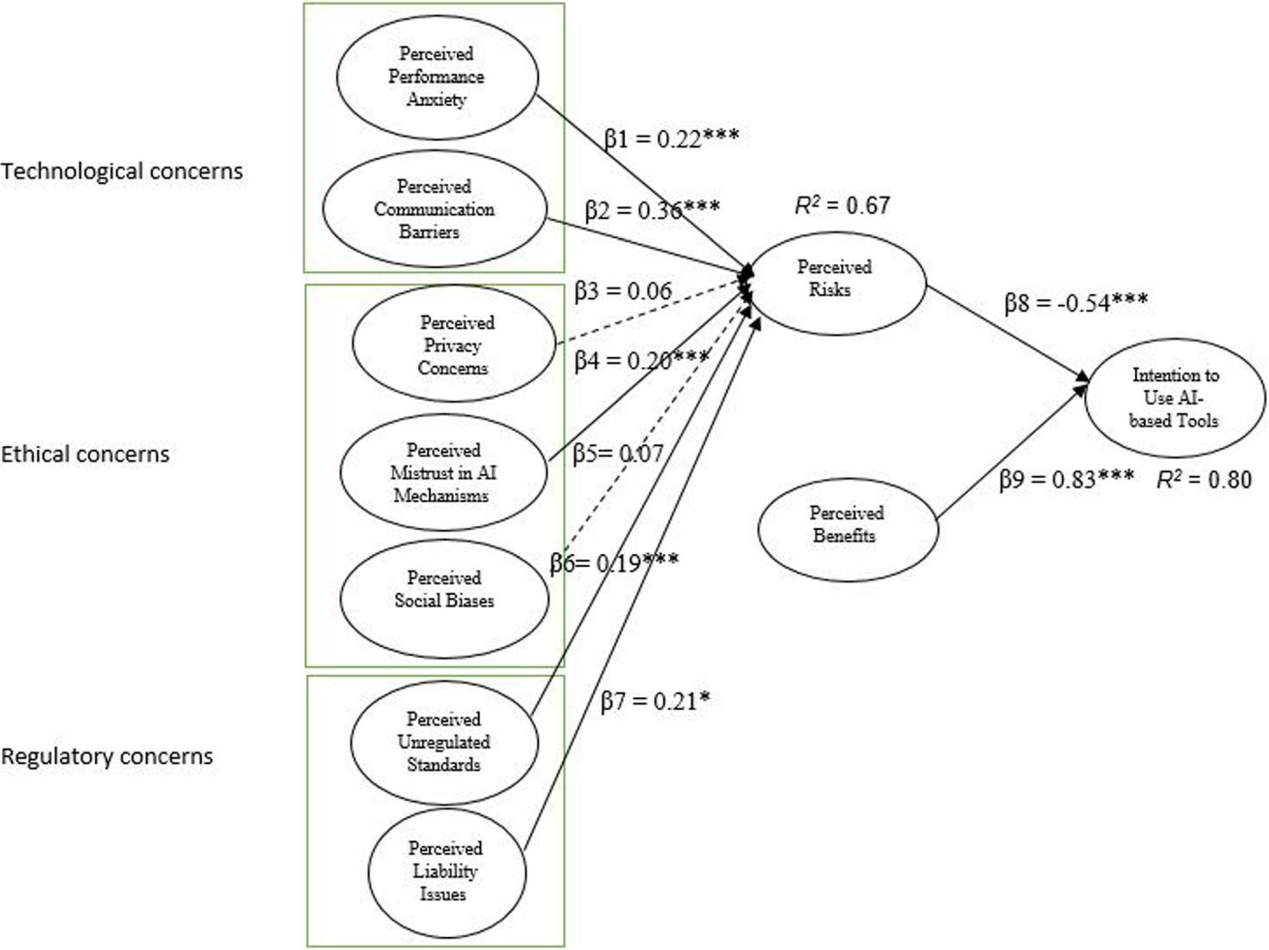
Model paths *P < 0.05, ***P < 0.001

IBM SPSS AMOS (version 26) was also used to test the hypotheses within a SEM framework. According to Ho [72], the goodness of fit statistics can evaluate the entire structural model and assess the overall fit. The findings indicated that the normed Chi-square value (χ2/df) was 2.23. The indices values for CFI = 0.91, NFI = 0.90, RFI = 0.93, and TLI = 0.90 were above 0.9 and the SRMR = 0.05 and RMSEA = 0.06 were below 0.08 [73]. The value of AGFI was 0.92 that exceeded 0.90. All these measures of fit were in the acceptable range, and only GFI = 0.84 were marginal. Based on Kline [74], at least four of the statistical values met the minimum recommended values, which supported a good fit between the hypothesized model and the observed data. Figure 2 displays the standardized path coefficients of the structural model under investigation.

The structural model was assessed by examining path coefficients. We performed bootstrapping (with 5000 bootstrap samples) to determine the significance of each path. The results of hypotheses testing are summarized in Table 5. With respect to technological concerns, the findings support H1 by showing the significant positive relationship between perceived performance anxiety and perceived risks (β = 0.22, *p* < 0.001). The findings provide enough evidence to support H2, which indicates that perceived communication barriers significantly reinforce perceived risks (β = 0.36, *p* < 0.001).

**Table 5:** Results of hypotheses testing

Hypothesis	Path	Standardized Coefficient	SE.	CR.	Results
H1	PPA → PR	0.22***	0.03	6.14	Supported
H2	PCB → PR	0.36***	0.05	6.03	Supported
H3	PPC → PR	0.06	0.05	1.06	Not- Supported
H4	PMT → PR	0.20***	0.048	4.28	Supported
H5	PSB → PR	0.07	0.10	0.69	Not-Supported
H6	PUS → PR	0.19***	0.04	3.85	Supported
H7	PL → PR	0.21*	0.10	2.09	Supported
H8	PR → INT	−0.54***	0.05	9.73	Supported
H9	PB → INT	0.83***	0.05	16.27	Supported

Perceived Risks *R*^*2*^: 0.67

Intention to Use AI-based Tools *R*^*2*^: 0.80

Table legend: *PPA* Perceived Performance Anxiety, *PSB* Perceived Social Biases, *PPC* Perceived Privacy Concerns, *PMT* Perceived Mistrust in AI Mechanisms, *PCB* Perceived Communication Barriers, *PUS* Perceived Unregulated Standards, *PL* Perceived Liability Issues, *PB* Perceived Benefits, *PR* Perceived Risks, *INT* Intention to Use AI-based Tools. ***P < 0.001, * *P* < 0.05

Regarding ethical concerns, support is not found for H3, which initially proposes that perceived privacy concerns significantly would contribute to perceived risks (β = 0.06, non-significant path). H4 is supported where higher perceived mistrust in AI mechanisms leads to greater perceived risks (β = 0.20, *p* < 0.001). H5, which posits that the perceived social biases would directly affect perceived risks, is not supported (β = 0.07, non-significant path). With regard to regulatory concerns, the analysis also demonstrates that individuals’ perception of unregulated standards positively influences perceived riks (β = 0.19, *p* < 0.001), and this positive linkage supports H6. The path coefficient of the relationship between perceived liability issues and perceived risks is significant, supporting H7 (β = 0.21, *p* < 0.05). The negative effect of perceived risks associated with AI on individuals’ intention to use AI-based tools is significant, supporting H8 (β = 0.54, *p* < 0.001). The findings also provide enough evidence to support H9 by indicating that the more perceived benefits associated with AI tools, the more likely individuals are to use AI-based devices for healthcare purposes (β = 0.83 and *p* < 0.001).

Finally, the variables used in the model explained 67% of the variance in perceived risks and 80% of the variance in individuals’ intention to use AI-based tools. The R^2^ scores reflect that the model provides relatively strong explanatory power to predict individuals’ adoption behaviors in the context of AI technology for healthcare purposes.

## Discussion

Due to the promising opportunities created by AI technology (such as better diagnostic and decision support), the main question is when AI tools will be part of routine clinical practice [75]. AI embedded in smart devices democratizes healthcare by bringing AI-enabled health services (such as AI-based CDS) into the homes of patients [30]. Nevertheless, some concerns related to the use of AI need to be addressed. Because of the sensitivity and novelty, the intention to use AI devices in healthcare may involve an alternative approach with stronger prediction power than existing models of technology acceptance. Furthermore, previous AI acceptance models are general and do not reflect particular professional contexts and characteristics that may raise multiple concerns [14]. We consider healthcare a sector with distinct, value-based nature and, thus, in need of a unique model for predicting the intention to use devices with AI-based CDS. We propose a model of AI clinical device acceptance that, by an extension of established risk-benefit factors [12], has a higher explanatory power to predict the intention to use AI devices in healthcare. As previous studies introduce several concerns and challenges with AI [25], the main focus of this model is to better explain and categorize factors affecting risk beliefs associated with AI. In our analysis, we demonstrate three categories of concerns with AI: technological, ethical, and regulatory. Technological concerns, which include two dimensions (perceived performance anxiety and perceived communication barriers), directly shape perceived risk with the use of AI. Ethical concerns consist of three dimensions (perceived privacy concerns, perceived mistrust in AI mechanism, and perceived social biases), and only trust factor emerge as a significant variable in the risk beliefs about AI tools. Regulatory concerns with two dimensions (perceived unregulated standard and perceived liability issues) directly contribute to risk beliefs associated with AI.

Findings suggest that three categories of individuals’ concerns have a significant impact on their assessment of the risks and benefits associated with AI-based CDS use; the stronger the concerns, the higher the risk perceptions, and the lower the benefit perceptions. Of the three categories, technological concerns (i.e., performance and communication) are found to be the most significant predictors of risk beliefs. This result is in line with previous findings that ambiguity about AI functional characteristics considerably influences risk perceptions associated with potential future use of AI [25]. There is a growing interest in research about AI-centric technologies, yet individuals have not integrated AI devices into many aspects of their lives [76]. We can argue that the general technical knowledge of the public about AI performance and how it works is still at an early stage. If AI-based devices gained more ground in everyday care work, people would possibly have more of a perspective about benefits and risks to accept the use of AI clinical tools.

Moreover, of the seven antecedents of risk beliefs, communication barriers are found to have the strongest relationship with risk perceptions. Based on this finding, individuals are concerned that AI devices may reduce human- aspects of relations in medical contexts. Therefore, they may lose face-to-face cues and personal interactions with physicians and find themselves in a more passive position for making health-related decisions. This finding is consistent with a study in the chatbot context (within the area of AI systems), which indicates that users have stronger feelings of co-presence and closeness when the chatbot uses social cues [77]. In the context of robot care, a study shows that when robots used in rehabilitation, they are viewed by patients as reducing human contact [78]. Developers need to add more interactive and entertaining social cues to devices with AI-based CDS features in healthcare to address the possible communication barriers between users and AI. For instance, AI-driven recommendations and assistance can be appealing if the device holds a promise of allowing users more time to interact with it to establish empathy.

Findings imply that perceived benefits from AI-based CDS significantly increase the intention to use AI technology in healthcare. In line with other studies, if users believe that AI-based devices can improve diagnostics, prognosis, and patient management systems, they become more likely to use them [79]. These results recommend that AI device developers highlight potential AI benefits in their marketing campaign to promote usability as well as the value of their AI tools and increase the use rate. Specific marketing strategies in medical AI device companies can be developed to enhance users’ state of awareness about how AI-based tools can suggest accurate care planning, reduce healthcare costs, and boost healthcare outcomes.

The results also indicate that the effects of benefit perceptions are higher than risk beliefs. This point may imply that AI-based CDS developers need to illustrate why AI-driven recommendations are suitable for healthcare tasks (i.e., highlighting benefits), and most importantly, they need to take action to address possible concerns (i.e., reducing risks). Thus, if developers attempt to persuade users by focusing on advantages, although concerns have not even been addressed, users are not likely to use AI tools for healthcare purposes. To find justification for the use of AI, an individual might be persuaded to adjust his/her value-based evaluations by viewing the change as creating more opportunities (health-related benefits) rather than threats (technological, ethical, and regulatory concerns). It should be mentioned that even though the main dependent variable in this study is the intention to use AI-based devices, we do not propose that an unconditional acceptance of AI clinical tools, is the ideal situation in healthcare. In contrast, we attempt to exhibit how value-based consideration is important when implementing AI devices in healthcare contexts. If the rejection of the use of medical AI is explained by huge and unaddressed technological, ethical, or regulatory concerns, there is not much sense in partially coping with these concerns by setting up the mandatory use of medical AI covering the whole patients. We propose that a successful rollout of AI-based devices in healthcare may need to be managed with the knowledge and consideration of potential users’ risk-benefit analysis.

The results confirm that perceived benefits and risk beliefs associated with AI predict higher intention to use AI-based CDS (R^2^ = 80%) compared to previous studies using extended acceptance models [3]. The data support all hypotheses developed in this study except for H3 and H5. Although previous conceptual studies suggest the importance of privacy and biases [19], our empirical research cannot provide evidence to confirm their effects. One possible explanation is the characteristics of our participants. Around 67% of our sample did not experience a severe online privacy breach (data loss, including personal, health, or financial information). Previous studies indicate that invasion of privacy in the past significantly influence risk beliefs [80]. Moreover, the lack of racial diversity in our sample (69.7%: white) may affect the direct relationship between perceived social biases and risk beliefs. According to previous studies, social biases are mostly believed against minority groups with insufficient data in AI datasets [81]. However, the overrepresentation of a majority group in our sample may cause that most of the respondents did not encounter societal discrimination due to unfair healthcare practice.

The second plausible justification is that respondents did not completely believe in AI performance (H1) and did not trust in AI’s predictive and diagnostic ability for treatment purposes (H4). Thus, we can argue that individuals still concern about the competency of AI-driven diagnostic options, treatment choices, prescription advice, and care planning. However, they may believe in the security system and technical safeguards embedded in AI devices to protect data privacy. Moreover, they may not trust AI competence, but they might have trusted in AI fair process. According to Komiak and Benbasat [82], people may not trust in expertise and knowledge of an information system (cognitive trust in competence), but they may trust in the integrity of the system (cognitive trust in integrity). Thus, based on the results, individuals may still not believe in the competence of the AI mechanism (such as accuracy of recommendations), but they think that AI provides unbiased and honest recommendations and advice to all social classes.

### Theoretical and practical contributions

This study makes some important contributions to the literature. Although several studies have examined a variety of AI-related topics in different contexts (for instance, in service delivery) [3], there is still a lack of understanding of how individuals’ perceptions toward AI-based CDS in healthcare are generated. Previous research mainly studies customers’ intention to use AI devices using existing technology acceptance theories (such as TAM, UTAUT) [83, 84]. Traditional acceptance models are originally developed to study behavioral intention to non-intelligent technologies and do not cover the characteristics of intelligent systems [85]. In the context of medical AI devices, individuals are likely to analyze whether an AI device can deliver the same level of or better service as physicians are expected to deliver. Thus, there is a need for potential users of AI-based tools within healthcare to understand possible outcomes and consequences (both opportunities and threats) of medical diagnosis and solutions created by an AI system. Due to the specificity of the healthcare field, we propose that a value-consideration approach would be a better alternative than technology acceptance models to examine why people will use AI systems in healthcare.

The findings suggest that the utilization of the value assessment approach may be more applicable to this context (R^2^ = 80%). Therefore, drawing on the value perspective, we propose that individuals’ final decision to accept the use of medical AI devices is also likely to be determined by their risk-benefit analysis. Our research also identifies the most critical concerns affecting individuals’ willingness to use AI devices in healthcare and validates the antecedents of risk beliefs. This study provides a conceptual framework for AI-based CDS acceptance that can be used by researchers to better examine AI-related topics in the other context.

This study has some practical implications for the diffusion of devices with AI-based CDS in healthcare. In this study, individuals’ positive perceptions toward AI-based devices can lead to a higher intention to use AI. Highlighting the performance benefits such as accuracy of diagnosis, reliability of data analysis, the efficiency of care planning, and consistency of treatments in communication with users and marketing materials may increase individuals’ intention to at least try services provided by AI devices in healthcare. Moreover, the concerns and challenges associated with AI have a substantial effect on the risk perceptions of people. If healthcare providers are not able to reduce concerns, some individuals may refuse using AI-based devices and may request traditional interactions with physicians. Even if hospitals decide to use AI devices as supportive services under the supervision of healthcare professionals, the mentioned concerns should be eliminated prior to the implementation of AI systems.

Addressing the concerns contributing to risk beliefs about AI is a priority. Society generally is yet to fully grasp many of the ethical and regulatory considerations associated with AI and big data [86]. Accountability involves a number of stakeholders such as AI developers, government agencies, healthcare institutions, healthcare professionals, and patient communities. Regulatory agencies, in cooperation with healthcare institutions, should establish normative standard and evaluation guidelines for the implementation and use of AI-based CDS in healthcare. The policies should clarify how AI-based devices will be designed and developed in healthcare to comply with accepted ethical principles (such as fairness and health equity). Regular audits and ongoing monitoring and reporting systems can be used to continuously evaluate the safety, quality, transparency, and ethical factors of AI-based services.

Devices with AI-based CDS should be designed in a way to respect patients’ autonomy and decision-making freedom. AI agents should not follow a coercive approach to force patients to make health-related decisions under pressure. Regulations should illuminate the role of patients in relation to AI devices so that they are aware of their position to refuse AI-base treatments where possible [87]. An important aspect that needs to be built into AI systems in healthcare is the transparency of AI algorithms so that the AI system doesn’t remain a black box to users. Technical education, health knowledge, and explicit informed consent should be emphasized in the AI implementation model to prepare patients for AI use. Training should target the patient community to ensure patients obtain enough information to make informed health decisions. Thus, if users understand the basics of AI devices, and what benefits and limitations they can bring to healthcare, they become more willing to accept AI use to obtain improved healthcare delivery. Under this circumstance, users will be active partners of AI tools rather than passive receivers of AI recommendations.

### Limitations and future studies

It should be mentioned that the study is based only on a sample of respondents drawn from the United States. Care work culture and technology use are different between countries. Moreover, the lack of racial diversity (69% were white) and age variety (66% were between 20 and 40) of the sample may be considered as a limitation in the generalizability of our results. Thus, it is recommended that future studies consider drawing samples with more representative subjects in wider geographical areas, including other developed countries and also developing countries where technological infrastructures and internet services are less developed than in the United States. Our study used an online survey to recruit participants digitally. Since a self-rated sample of participants on MTurk was used, there is a small chance that some respondents were not completely aware of AI technology and formed their mental construal of the IT artifact. Therefore, we suggest that further studies use a different method to ensure that subjects are knowledgeable about medical AI. For instance, future research can recruit informed patients who are directly referred by the providers using patient self-management tools such as wearable devices with embedded AI. Besides, our study used an online survey to recruit participants digitally, which might induce sample selection bias. Thus, we only considered individuals who could access the computer, mobile devices, and the Internet to participate in the online survey. Future studies can use other data collection means and sampling strategies to reach out to a sample that is generalizable to a wide range of healthcare consumers. Moreover, control variables (such as familiarity with AI devices, computer skills, health literacy, technical knowledge about AI) were rated by a self-assessment scale. Future research could use standard scales (with validated items) to measure them.

This study can also serve as a starting point for further empirical studies in the context of individual adoption of AI clinical devices. In this study, we used the general concept of AI, and no specific type of AI clinical tools was examined. Value assessments may have different underlying objectives, depending on the type of AI device. For instance, it would be interesting to investigate how alternative AI device brands influence risk beliefs and, in turn, affect intention. Moreover, we defined AI devices as the tool that consumers can voluntarily choose to use for healthcare management. Another promising research avenue would be to examine public perspectives in other healthcare contexts, e.g., when AI tools are implemented and used in hospitals and healthcare professionals recommend that patients use AI devices. Or for example, a follow-up study is needed to examine users’ value perceptions in a situation that the use of the AI devices may be a mandatory part of performing diagnosis and completing patient treatments.

Future studies can also extend our findings to examine the acceptance of AI devices among individuals with chronic physical diseases or mental disorders and analyze the plausible differences. Since our study focuses on patients, a follow-up study could investigate the hypotheses with groups of nurses, physicians, therapists, etc., to examine whether the same factors are associated with technology acceptance of AI-based medical devices when it comes to healthcare professionals. We also propose that AI researchers conduct further studies from the perspectives of hospital management. Thus, other factors, such as economic and organizational challenges, should be added to our model as new categories of concerns. Finally, although the predictive power of the model is acceptable (80%), it might be useful if future studies can add other factors to the model to increase exploratory power. For instance, social influence can be integrated into the model since the effect of this variable is particularly important when individuals do not have sufficient knowledge about the technology to make an informed decision.

## Conclusions

Disruptive advances in technology inevitably change societies, communications, and working life. One of the fundamental changes that could impose significant effects on healthcare is the widespread implementation of AI devices. AI technology is an integral element of many organizations’ business models, and it is a critical strategic component in the plans for many sectors of business, such as healthcare institutions. Implementing advanced information systems (such as AI) in healthcare requires an in-depth understanding of the factors associated with technology acceptance among groups of stakeholders. One of the most important stakeholders of devices with AI-based CDS is patients. Due to the special characteristics of the healthcare sector, the implementation of AI devices should be conducted with several necessary considerations. From the public perspective, using AI devices is to endorse them. Our model suggests that during a decision-making process, individuals go through a stage of appraisal, including evaluating the value of AI-based CDS (benefits versus risks). If technological, ethical, ad regulatory concerns are not analyzed, rationalized, and resolved accordingly, people may not only use them but also view AI devices as a threat to their healthcare. AI device developers need to highlight potential benefits from AI technology and address different dimensions of concerns to justify the purchase and use of an AI tool to the public. Healthcare regulatory agencies need to clearly define the right and the responsibility of healthcare professionals, developers, programmers, and end-users to demonstrate acceptable approaches in the use of AI devices.

## Supplementary information

**Additional file 1 Appendix:** Measurement instrument.

## Data Availability

A significant part of the data analyzed in this study is included in this published article. The datasets used and analyzed during the current study are available from the corresponding author on reasonable request.

## Abbreviations

AI: Artificial Intelligence
CDS: Clinical Decision Support
FDA: Food and Drug Administration
TAM: Technology Acceptance Model
UTAUT: Unified Theory of Acceptance and Use of Technology
TPB: Theory of Planned Behavior
MTurk: Amazon’s Mechanical Turk
SEM: Structural Equation Modeling
CFA: Confirmatory Factor Analysis
AVE: Average Variance Extracted
VIF: Variance Inflation Factor
