# Supplementary material for: Use of AI-based tools for healthcare purposes: a survey study from consumers’ perspectives
Source: BMC Med Inform Decis Mak. 2020 Jul 22;20:170. doi: 10.1186/s12911-020-01191-1 (PMC7376886; doi:10.1186/s12911-020-01191-1)
Supplement: Supplementary file 1 — Additional file 1 Appendix: Measurement instrument. [file 12911_2020_1191_MOESM1_ESM.docx]

**Appendix:**

Measurement instrument

| **Construct** | **Item** | **Wording** |
| --- | --- | --- |
| Perceived performance anxiety | PRA1 | I am concerned that the mechanisms used by AI-based devices may lead to inaccurate predictions |
|  | PR2 | I am concerned that the mechanisms used by AI-based devices may result in medical errors |
|  | PR3 | I am concerned that treatments provided by AI devices may be incomplete |
|  | PR4 | I am concerned that the predictive models of AI-based tools may malfunction |
|  | PR5 | I am concerned that the medical decisions made by AI devices may be inadequate |
| Perceived social biases | PSB1 | I am concerned that the AI-based devices may overestimate or underestimate health risks in a certain patient population (e.g., people with insufficient data in AI datasets) |
|  | PSB2 | I am concerned that data used in the AI devices may lead to societal discrimination to a certain patient group (e.g., minority groups) |
|  | PSB3 | I am concerned that AI-based tools used in healthcare may be unfair to a certain group of population (e.g., people with poor access to health care) |
|  | PSB4 | I am concerned that AI devices could lead to morally flawed practices in health care |
|  | PSB5 | Overall, I am concerned that the possibility of biases by AI devices to certain groups of the population is high |
| Perceived privacy concerns | PPC1 | I think using AI-based applications helps health care entities collect too much personal information from me |
|  | PPC2 | I think in this case, I am concerned that health care entities use my health information for other purposes without my knowledge and authorization |
|  | PPC3 | In this case, I am concerned that my health information will be shared with other entities without my explicit consent |
|  | PPC4 | In this case, I am concerned that unauthorized people will have access to my health information |
|  | PPC5 | In this case, I am concerned about the privacy of my health information during AI-based health practices |
|  | PPC6 | In this case, I am concerned my health information would be sold to others without my permission |
| Perceived mistrust in AI mechanisms | PMT1 | I trust in the AI-based clinical tools used for healthcare delivery |
|  | PMT2 | I trust in the AI algorithms used in the healthcare |
|  | PMT3 | I trust in AI 's predictive and diagnostic ability for treatment purposes |
|  | PMT4 | I trust in the accuracy and predictive powers of current AI algorithmic models used in the medical context |
|  | PMT5 | I trust that AI-based tools can adapt to specific and unforeseen medical situations. |
| Perceived communication barriers | PCB1 | I am concerned that AI tools may eliminate the contact between healthcare professionals and patients |
|  | PCB2 | I am concerned that AI tools may reduce conversation between physicians and patients |
|  | PCB3 | I am concerned that AI devices may decrease human- aspects of relations in the medical contexts |
|  | PCB4 | I am concerned that by using AI devices, I may lose face-to-face cues and personal interactions with physicians |
|  | PCB5 | I am concerned that by using AI devices, I may be in a more passive position for making medical decisions |
| Perceived unregulated standard | PUS1 | I am concern that special policies and guidelines for AI tools are not transparent yet |
|  | PUS2 | I am concerned that the safety and efficacy of AI medical tools are not regulated clearly |
|  | PUS3 | I am concerned that regulatory standards to assess AI algorithmic safety are yet to be formalized |
|  | PUS4 | I am concerned that appropriate regulatory and accreditation system regarding AI-based devices is not in place yet |
|  | PUS5 | I am concerned about the lack of clear guidelines to monitor the performance of AI tools in the medical context |
| Perceived liability issues | PL1 | I am concerned because it is not clear who is responsible when errors result from the use of AI clinical tools |
|  | PL2 | I am concerned about the liability of using AI-based services for my healthcare |
|  | PL3 | I am concerned because it is not clear who becomes responsible if AI-based tools offer wrong recommendations |
|  | PL4 | I am concerned because it is unclear where the lines of responsibility begin or end when AI devices guide clinical care |
|  | PL5 | I am concerned because it is not clear who is responsible if appropriate AI-recommended treatment options are mistakenly dismissed |
|  | PL6 | Overall, I am concerned that the use of AI clinical tools for clinical purposes increases my liability |
| Perceived risks  (very low/very high) | PR1 | The risk of using AI-based tools for medical purposes is |
|  | PR2 | The degree of uncertainty associated with the use of AI clinical tools is |
|  | PR3 | The potential loss associated with the use of AI devices is |
|  | PR4 | The likelihood of unexpected problems with the use of AI devices is |
|  | PR5 | Overall, the chance of adverse consequences associated with the use of AI-based tools for healthcare purposes is |
| Perceived benefits | PB1 | I believe AI-based services can improve diagnostics |
|  | PB2 | I think AI-based devices can enhance prognosis |
|  | PB3 | I believe AI-based devices can advance patient management systems |
|  | PB4 | I believe AI-based tools can suggest accurate care planning |
|  | PB5 | I think AI-based services can recommend reliable treatment options |
|  | PB6 | I think AI-based tools can reduce healthcare costs |
|  | PB7 | Overall, I think AI-based devices can boost healthcare outcomes |
| Intention to use AI-based tools | INT1 | I agree to use AI-based tools for clinical purposes |
|  | INT2 | Using AI-based tools for healthcare purposes is something I would consider |
|  | INT3 | I would like to use AI-based devices to manage my healthcare |
|  | INT4 | In the future, I am willing to use AI-based services for diagnostics and treatments |
|  | INT5 | I am very likely to use recommendations provided by AI-based tools for care planning |
